# A novel chlorination-induced ribonuclease YabJ from *Staphylococcus aureus*

**DOI:** 10.1042/BSR20180768

**Published:** 2018-10-15

**Authors:** Hyo Jung Kim, Ae-Ran Kwon, Bong-Jin Lee

**Affiliations:** 1 Research Institute of Pharmaceutical Sciences, College of Pharmacy, Seoul National University, Gwanak-gu, Seoul 151-742, Korea; 2 Department of Herbal Skin Care, College of Herbal Bio-industry, Deagu Haany University, Gyeongsan 712-715, Korea

**Keywords:** chlorination, post translational modification, ribonuclease, RidA, YabJ, YjgF family

## Abstract

The characteristic fold of a protein is the decisive factor for its biological function. However, small structural changes to amino acids can also affect their function, for example in the case of post-translational modification (PTM). Many different types of PTMs are known, but for some, including chlorination, studies elucidating their importance are limited. A recent study revealed that the YjgF/YER057c/UK114 family (YjgF family) member RidA from *Escherichia coli* shows chaperone activity after chlorination. Thus, to identify the functional and structural differences of RidA upon chlorination, we studied an RidA homolog from *Staphylococcus aureus*: YabJ. The overall structure of *S. aureus* YabJ was similar to other members of the YjgF family, showing deep pockets on its surface, and the residues composing the pockets were well conserved. *S. aureus* YabJ was highly stable after chlorination, and the chlorinated state is reversible by treatment with DTT. However, it shows no chaperone activity after chlorination. Instead, YabJ from *S. aureus* shows chlorination-induced ribonuclease activity, and the activity is diminished after subsequent reduction. Even though the *yabJ* genes from *Staphylococcus* and *Bacillus* are clustered with regulators that are expected to code nucleic acid-interacting proteins, the nucleic acid-related activity of bacterial RidA has not been identified before. From our study, we revealed the structure and function of *S. aureus* YabJ as a novel chlorination-activated ribonuclease. The present study will contribute to an in-depth understanding of chlorination as a PTM.

## Introduction

Post-translational modification (PTM) is a biochemical process where amino acids of proteins are covalently modified after translation. PTMs are critical regulators of proteins’ physiochemical properties, functions, and interactions with other proteins or nucleic acids. They are rapid, specific, and highly controlled modifications involved in cellular processes, such as enzyme activity, protein turnover, and protein localization [1]. More than 200 different types of PTMs have been identified, such as phosphorylation, glycosylation, ubiquitination, nitrosylation, methylation, acetylation, lipidation, and proteolysis [2]. Although PTMs play fundamental roles in the regulation of numerous pathways of cells, many types of PTMs still remain to be elucidated. Furthermore, various studies about different types of PTMs are reported each year, including chlorination [3]. Chlorination activates chaperone activity of Hsp33 through partial unfolding of its C-terminal redox-switch domain [4]. β-lactoglobulin, α-amylase, and BSA are other examples of proteins that gain chaperone activity after chlorination [5]. A recent study also revealed that *Escherichia coli* RidA from the YjgF/YER057c/UK114 family (YjgF family hereafter) exhibits chlorination-induced chaperone activity [5]. Given that chlorination can activate protein functions, it suggests that chlorination is an important PTM that is closely involved with cellular networks [6–8].

The YjgF family is divided into eight subfamilies according to NCBI Conserved Domain Database: RidA and Rid1–Rid7 (see cd00448: YjgF_YER057c_UK114_family). An organism may have RidA and other Rid family proteins [9]. Several crystal structures of YjgF family members have been solved, showing common features. Most are ball-like trimers with a hole in the center, formed by β-strands, with one pocket on each monomer, formed by loops. The six footprint residues (Tyr^17^, Ser^30^, Asn^88^, Arg^105^, Cys^107^, and Glu^120^ in *E. coli* RidA) are positioned in the pocket on each monomer and are used as a position-specific scoring matrix to differentiate the Rid subfamilies. These six footprint residues are expected to be functional sites. For example, the Rid4–Rid7 subfamilies, which lack Arg^105^, do not show any deaminase activity. Almost all other subfamily members have deaminase activity that removes reactive enamine/imine intermediates during branched chain amino acid biosynthesis pathways and accelerates the release of ammonia [10,11]. Therefore, the family was recently suggested to be renamed Rid (reactive intermediate/imine deaminase). The typical RidA subfamily members are found in diverse organisms, such as *Bacillus subtilis* (YabJ), *Drosophila melanogaster* (DUK114), and *Rattus norvegicus* (L-PSP). Besides deamination, other functions of the RidA subfamily include maintenance of the mitochondrial genome (*Saccharomyces cerevisiae*, YER057C), translation inhibition (human hp14.5), and chaperone activity (*E. coli* RidA and *D. melanogaster* DUK114) [9,12].

Gene clustering in prokaryotes indicates a functional relationship, and bacterial RidA subfamily genes are usually clustered with pyrimidine or arginine metabolism genes [13]. Unlike other bacterial RidA subfamily member genes, those from *Bacillus* and *Staphylococcus* species show unique genetic profiles [14]. The gene encoding RidA in *Bacillus* and *Staphylococcus* is clustered with *spoVG*, forming the *yabJ-spoVG* bicistronic operon. In *Bacillus* and *Staphylococcus* species, RidA is encoded from the gene *yabJ*; therefore, the RidA proteins encoded by these genes are subsequently termed YabJ (_BS_YabJ and _SA_YabJ hereafter). *yabJ* and *spoVG* transcription depends heavily on σ^B^, a large regulon of genes involved in many different cellular processes [15]. Although σ^B^ controls the expression of multiple genes, including virulence factors, gene regulators, and antibiotic resistance, the σ^B^ promoter is not directly responsible for this regulation. Rather, σ^B^-dependent regulation is controlled by downstream regulatory elements such as the *yabJ-spoVG* operon. Indeed, inactivation of the *Staphylococcus yabJ-spoVG* operon causes a reduction in nuclease, protease and lipase activity, and resistance to antibiotics [16,17]. The two proteins encoded from the bicistronic operon are expected to work as regulators by interacting with nucleic acids. Even though _BS_YabJ represses the function of *purR*, which encodes adenylosuccinate synthetase, the mechanism has not yet been revealed [18].

Here, we elucidated the crystal structure of _SA_YabJ from *Staphylococcus aureus* Mu50 and its function as a ribonuclease after chlorination. Because *yabJ* is clustered with another DNA-binding protein gene in the *yabJ-spoVG* operon, and using the domain prediction for YabJ in the UniProtKB database, _SA_YabJ is predicted to have a nuclease function. Although the structures of many RidA homologs are solved, only L-PSP has shown ribonuclease activity [19]. The hp14.5 has translation inhibition activity, although the mechanism is still unclear [20]. Considering that mammalian RidA homologs primarily show nucleic acid-related activity amongst many homologs and liver perchloric acid extracted L-PSP and trichloroacetic acid extracted hp14.5 show higher activity than recombinant L-PSP and hp14.5, YabJ is predicted to go through chlorination as a PTM. To gain further insight into the function of _SA_YabJ, we verified whether chlorination was possible and ribonuclease activity assays were conducted. Chlorination was reversible with a reducing agent, and _SA_YabJ shows ribonuclease activity after chlorination and loses activity when reduced. In addition, we identified important residues for the ribonuclease activity by site-directed mutagenesis. The present study presents a ribonuclease that is activated following chlorination and the novel discovery of the nuclease function of YabJ. Our structural and functional research regarding chlorination of _SA_YabJ will promote a better understanding of this type of PTM.

## Experimental

### Cloning, expression, and purification

An expression plasmid for SAV0497 (*yabJ*) from *S. aureus* Mu50 was constructed using ligation-independent cloning (LIC) as previously described [21–23]. The resulting construct has 17 additional residues (MHHHHHHENLYFQGAAS) that encode an N-terminal hexa-histidine tag, TEV cleavage site, and GAAS residues for LIC cloning. To prepare mutants (C103A, pocket-forming sites mutant: Y16H18K39R101C103K111A), the EZchange Site-directed Mutagenesis kit (Enzynomics, Korea) was used. The sequences of the cloned genes were confirmed by DNA sequencing (results not shown). The recombinant plasmids were transformed into *E. coli* BL21 (DE3). Cells were grown at 37°C in LB medium supplemented with ampicillin (50 μg/ml) until the OD_600_ reached 0.5. Recombinant protein expression was induced by the addition of IPTG to 0.5 mM, and the cells were allowed to grow for an additional 4 h at 37°C. The cells were harvested by centrifuging at 4500 ***g*** at 4°C. For each protein, cell pellet was resuspended in lysis buffer (50 mM Tris/HCl, pH 7.5, and 500 mM NaCl) and disrupted using an Ultrasonic processor (Cole-Parmer, U.S.A.) at 4°C. The cell lysate was centrifuged at 20000 ***g*** for 1 h at 4°C. The cleared supernatant was purified by binding to an Ni-NTA (Ni^2+^-nitrilotriacetate) affinity column (Qiagen, Germany; 3 ml of resin per liter of cell lysate) and eluted with binding buffer containing 200 mM imidazole. Further purification and buffer exchange were achieved by size-exclusion chromatography using a Superdex 75 (10/300 GL) column (GE Healthcare Life Sciences, U.S.A.) that was previously equilibrated with 50 mM Tris/HCl, pH 7.5, and 200 mM NaCl. The purity of recombinant _SA_YabJ was estimated to be over 95% by SDS/PAGE. The purified _SA_YabJ was concentrated to 10 mg/ml by ultrafiltration in 10000 Da molecular-mass cut-off spin columns (Millipore, U.S.A.). The absorbance at 280 nm was measured, and the calculated molar absorption coefficient of 5960 M^−1^.cm^−1^ (Swiss-Prot;http://www.expasy.org) was employed to determine the protein concentration.

### Crystallization, X-ray data collection, and structure determination

Crystals of _SA_YabJ were grown by the hanging-drop vapor diffusion method at 293 K using 24-well VDX plates (Hampton Research, U.S.A.). Initial crystallization conditions were established using screening kits from Hampton Research (Crystal Screens I and II, Index, PEG/Ion, and MembFac) and from Emerald BioSystems (Wizard I, II, III, and IV). For the optimal growth of the _SA_YabJ crystals, each hanging drop was prepared on a siliconized coverslip by mixing 1 μl of 10 mg/ml protein solution and 1 μl of precipitant solution (25% (w/v) PEG3350 and 100 mM Tris/HCl, pH 8.5) and this drop was equilibrated against a 1-ml reservoir of precipitant solution. The condition yielded needle-shaped crystals that grew to dimensions of 1.2 mm × 0.4 mm × 0.4 mm in 10 days. For crystal freezing, the crystals were transferred to a cryoprotectant solution with 30% glycerol in the crystallization conditions for several minutes before being flash frozen in a stream of nitrogen gas at 100 K. Diffraction data were collected on ADSC Quantum 315r CCD detector system (Area Detector Systems Corporation, U.S.A.) at the BL-5C experimental station of the Pohang Light Source, Korea. The crystal was rotated by 1° for each image, and the raw data were processed and scaled using the program suite HKL2000 [24]. Further data analysis was carried out using the CCP4 suite [25]. The crystal belonged to space group P2_1_ and contained six molecules per asymmetric unit. Data collection statistics are summarized in Table 1.

**Table 1.** Crystallographic data collection and refinement statistics

	_SA_YabJ
**Data collection**	
Beamline	PAL-5C
Wavelength (Å)	0.98
Resolution range^1^ (Å)	40.00–1.75 (1.78–1.75)
Space group	P2_1_
Unit cell parameters (Å)	a = 47.12
	b = 83.22
	c = 89.36
Observations (total/unique)	277503/133402
Completeness (%)	97.4 (95.1)
CC_1/2_	0.98 (0.92)
R_sym_^2^	5.6 (20.4)
I/sigma	42.6 (6.9)
**Refinement**	
R_work_^3^ (%)	17.4
R_free_^3^ (%)	21.2
Protein atoms	5838
Water molecules	499
Average *B* value (Å^2^)	25.0
r.m.s.d. bond (Å)	0.006
r.m.s.d. angle (°)	0.801
**Ramachandran analysis (%)**	
Favored region	95.6
Allowed region	4.4
Outliers	0.0

1 Numbers in parentheses indicate the statistics for the last resolution shell.

2 ${\text{R}}_{\text{sym}}=\sum (\left|I_{hkl}-<I_{hkl}>\right|/\sum <I_{hkl}>$, where *I_hkl_* = single value of measured intensity of *hkl* reflection, and <*I_hkl_*> = mean of all measured value intensity of *hkl* reflection.

3 ${\text{R}}_{\text{work}}=\sum \left|F_{obs}-F_{calc}\right|/\sum F_{obs}$, where *F_obs_* = observed structure factor amplitude, and *F_calc_* = structure factor calculated from model. R_free_ is computed in the same manner as R_work_, but from a test set containing 5% of data excluded from the refinement calculation.

To determine the structure of _SA_YabJ, molecular replacement was used with the program MolRep within the CCP4 suite using the structure of homolog YabJ from *B. subtilis* (PDB code: 1QD9) as a search model [26]. The sequence identity of the two proteins is 59%. Refinement of the crystal structure was done through iterative cycles of model building using COOT, followed by refinement of the models with Refmac5 and phenix.refine [27–32]. A 5% portion of the data was set aside before the refinement for the R_free_ calculations [33]. Solvent molecules became apparent in the later stages of refinement. Refinement was pursued until no further decrease in R_free_ was observed. The final models exhibited good stereochemical geometry when the overall geometry was validated with PROCHECK [34]. Refinement statistics are summarized in Table 1. Structural alignments were carried out using the program PyMOL (http://www.pymol.org) and USCF chimera (http://www.cgl.ucsf.edu/chimera), which were then used for the construction and generation of all figures [35]. Protein interfaces, surfaces, and assemblies were calculated using the PISA server at the European Bioinformatics Institute (http://www.ebi.ac.uk/pdbe/prot_int/pistart.html) [36].

### Protein chlorination and oxidation

Hypochlorous acid (HOCl) was prepared from sodium hypochlorite (NaOCl) (Sigma, U.S.A.). Ten percent of the NaOCl solution was diluted to 200 mM in 50 mM Tris/HCl, pH 7.5. For complete chlorination of _SA_YabJ, 1 mM _SA_YabJ was incubated for 30 min at room temperature in HOCl with a molar ratio of _SA_YabJ:HOCl of 1:10. After treatment, excess HOCl was removed by extensive dialysis with 50 mM Tris/HCl, pH 7.5. The control solution consisted of the same _SA_YabJ with a buffer, which was incubated under the same conditions. The chlorinated _SA_YabJ (1 mM) was processed with 10 mM DTT to reduce the chlorinated state. Subsequently, DTT was removed by extensive dialysis with 50 mM Tris/HCl, pH 7.5.

For complete oxidation of _SA_YabJ, 1 mM _SA_YabJ was incubated for 30 min at room temperature in H_2_O_2_ (Sigma, U.S.A.) with a molar ratio of _SA_YabJ:H_2_O_2_ of 1:10. Excess H_2_O_2_ was removed by extensive dialysis with 50 mM Tris/HCl, pH 7.5. The chlorination and oxidation state of _SA_YabJ was determined by MS and native PAGE.

## PAGE

SDS/PAGE was conducted according to the Laemmli method using a 12% (w/v) polyacrylamide gel [37]. The samples were treated with 1% (w/v) SDS and 5% (v/v) 2-mercaptoethanol at 100°C for 5 min before electrophoresis in a vertical Mini Gel system (Bio-Rad, U.S.A.). The proteins were stained with Coomassie Brilliant Blue R250 (Thermo Scientific, U.S.A.). Additionally, for the separation of native _SA_YabJ, native PAGE was performed and analysis was conducted using 12% (w/v) polyacrylamide gel without either SDS or 2-mercaptoethanol. Native PAGE was performed in 25 mM Tris/HCl, pH 8.3, and 192 mM glycine. The staining was performed as for SDS/PAGE.

### Mass spectroscopy

Mass analysis was performed on a nano-HPLC system (Dionex Ultimate 3000 RSLCnano System, Thermo Scientific, U.S.A.) coupled with a hybrid quadrupole-orbitrap mass spectrometer (Q-Exactive, Thermo Scientific, U.S.A.) at the National Instrumentation Center for Environmental Management (NICEM, Seoul National University, Korea). Protein samples (10 μl) were loaded on to a C_8_ reverse-phase column (INNO5, Young Jin Biochrom, Korea). A room temperature gradient from 0.1% formic acid in water (solvent A) to 0.1% formic acid in acetonitrile (solvent B) was used for HPLC. The total run time for each sample was 20 min. The molecular mass of the protein was generated from several multiply charged peaks using the Xcalibur 2.2 software (Thermo Scientific, U.S.A.).

### CD

CD spectra were collected using a Chirascan series spectrometer equipped with a temperature controller (Applied Photophysics, U.K.) [38]. The protein samples were prepared in 50 mM Tris/HCl, pH 7.5 and 200 mM NaCl. The CD spectra were recorded with a step size of 1.0 nm, a bandwidth of 1 nm, and an averaging time of 2 s. Measurements were performed in a 1-mm path length quartz SUPRASIL cell (Hellma, Germany) using 10 μM concentration of _SA_YabJ at room temperature. Three scans were applied continuously and the data were averaged. The CD spectra were smoothed and processed after blank subtraction using Pro-Data Viewer software (Applied Photophysics, U.K.). The change in molar ellipticity [θ] was calculated using the following equation, where θ is in millidegrees, path length (l) is in millimeters, and C is the molar concentration of protein:

[θ] = θ/(1   ×   C ×   Number of residues)

### Chaperone activity assay

To monitor the chaperone activity, citrate synthase was employed as a substrate [39,40]. Initially, to identify foldase chaperone activity, 75 μg of citrate synthase (Sigma, U.S.A.) was mixed with a solution of 100 mM Tris/HCl, pH 8.0, 20 mM DTT, and 6 M guanidinium chloride. The citrate synthase mixture (75 μg of citrate synthase, 100 mM Tris/HCl, pH 8.0, 6 M guanidinium chloride, and 20 mM DTT) was incubated for 1 h at 25°C; consequently, the citrate synthase in this solution was denatured. After incubation, refolding of citrate synthase was achieved by 100-fold dilution with a solution of 100 mM Tris/HCl, pH 8.0 containing 5 μM _SA_YabJ. Hsp31 from *S. aureus* was used for positive control and buffer containing citrate synthase was used for negative control [41]. The diluted solution was mixed with 100 mM Tris/HCl, pH 8.0, 1 mM DTNB (5,5′-dithiobis [2-nitrobenzoic acid]), 0.2 mM MnCl_2_, 0.4 mM oxaloacetic acid, and 0.3 mM acetyl-CoA to detect the activity of citrate synthase. After mixing, only the active refolded enzyme could catalyze the breakdown of acetyl-CoA into the acetyl group and CoA. The CoA reacts with DTNB, which acts as a coloring agent, and this produces a yellow TNB (5-thio-2-nitrobenzoic acid)-CoASH compound that is detectable at 412 nm using a Multi-Mode microplate reader (SpectraMax M5e, Molecular Devices, U.S.A.) [42].

An aggregation prevention assay for chaperone activity was performed using citrate synthase mentioned above. Citrate synthase (30 μM) (Sigma, U.S.A.) was denatured in 4.5 M guanidinium chloride, 50 mM Tris/HCl, pH 7.5 at room temperature overnight. A total of 0.3 μM citrate synthase aggregation was monitored in 50 mM Tris buffer (pH 7.5) containing 5 μM _SA_YabJ at 30°C. Hsp31 from *S. aureus* was used as a positive control. The emission and excitation wavelengths were set at 360 nm [5].

### Ribonuclease activity assay

Purified, recombinant _SA_YabJ was incubated at 37°C for 30 min with the nucleic acid substrates: dsDNA, ssDNA, and ssRNA. Nucleic acid substrates (30 μM) were mixed with 0, 30, 60, and 120 μM _SA_YabJ in 50 mM Tris/HCl, pH 7.5 and 150 mM NaCl to a final volume of 5 μl. The total solutions were loaded on to 0.8% agarose gels in TBE buffer, and the results were visualized using a Gel Doc (Bio-Rad, U.S.A.). The nucleic acid used in the present study was an expected promoter of the *spoVG* gene of the *yabJ-spoVG* operon. In detail, oligomers used for the agarose gel assay were AATAAAACAGAGAGATATATACTATAGGG (29 nts) as ssDNA, dsDNA for the same sequence, and AAUAAAACAGAGAGAUAUAUACUAUAGGG (29 nts) as ssRNA [43].

The ribonuclease activity assay was complemented by a fluorescence assay using the RNase Alert assay kit (IDT, U.S.A.) [44,45]. A fluorophore is covalently linked to one end and a quencher on the other end of a synthetic RNA. When a ribonuclease cleaves the synthetic RNA of a fluorophore–quencher pair, the separated fluorophore emits green fluorescence at 520 nm following excitation at 490 nm. _SA_YabJ and mutants (C103A and pocket-forming sites mutant) were incubated at 37°C with the substrate (50 pmol) in 20 mM Tris/HCl, pH 7.0 and RNaseAlert buffer for 30 min. The resulting fluorescence (RFU) was monitored in a continuous assay format using a Multi-Mode microplate reader (SpectraMax M5e, Molecular Devices, U.S.A.).

## Results

### Crystal structure of *S. aureus* YabJ

**Figure 1:**
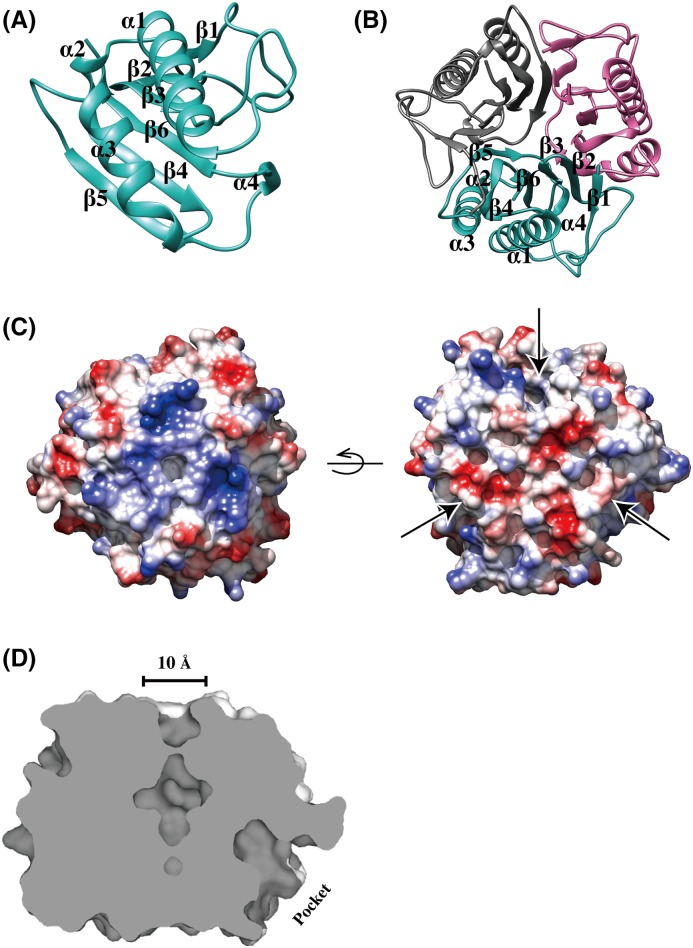
Crystal structure of *S. aureus* YabJ (**A**) Ribbon representation of the _SA_YabJ monomer. _SA_YabJ shows a β-sheet with two α-helices packed against it. The two 3_10_ helices form links between two layers. The topology of _SA_YabJ is β1-β2-β3-α1-α2-β4-α3-β5-α4-β6. (**B**) The _SA_YabJ trimer is shown in a ribbon representation from top view. Chain A is colored in cyan, chain B is colored in magenta, and chain C is colored in gray. β-strands are packed in the center area and α-helices cover the surface area. (**C**) Potential surface charge of the crystal structure of _SA_YabJ, calculated with UCSF Chimera, where surfaces are colored between −10 kcal/mole (red) and +10 kcal/mole (blue) (1 kcal = 4.184 kJ). The one side of the hole is positively charged (from top view) and the other side is negatively charged (from bottom view). Three pockets of the surface are indicated as arrows. (**D**) Cross-section of _SA_YabJ. A 10 Å-diameter hole is located in the middle and the deep pocket of one monomer is shown.

The 1.75-Å crystal structure of _SA_YabJ has clear electron density for 125 amino acids. Each _SA_YabJ monomer consists of a β-sheet and two α-helices. Six β-strands, β1 (residues 2–4), β2 (residues 19–22), β3 (residues 25–28), β4 (residues 70–77), β5 (residues 100–105), and β6 (residues 115–122) are aligned in a single sheet, and two α-helices, α1 (residues 45–63) and α3 (residues 80–92) are packed against the β-sheet. Additionally, two 3_10_ helices are observed in the structure: α2 (residues 67–69) is positioned at the end of α1 and α3 and packed against the β-sheet, and α4 (residues 110–112) are located between a loop region and to the next monomer’s β-sheet (Figure 1A).

The crystal structure reveals that _SA_YabJ exists as a compact homotrimer, which is consistent with the results of size-exclusion chromatography (Figures 1B and 3A). The ball-like trimeric structure forms a hole in the center area with a diameter of approximately 10 Å, with ∼20 water molecules positioned in the interior. The interface buries ∼988 Å^2^ of surface per subunit (13.7% of the subunit surface). The β-strands (β2–β6) form a central barrel with a hollow center that does not completely permeate the protein, and all α-helices are located around the perimeter, solvent exposed (Figure 1B). On its trimeric interface, there are numerous residues that contribute hydrogen bonds between the subunits, including Ser^18^, Thr^21^, Asn^24^, Ser^29^, Ser^69^, Asn^81^, Lys^98^, Arg^101^, Ser^102^, and Glu^116^. Lys^72^ and Glu^118^, Lys^122^ and Asn^23^, and Asn^81^ and Arg^108^ form charge–charge interactions with adjacent monomers.

The surface electrostatic potential map shows that positively charged residues predominate at the entry of the center cavity, and negatively charged residues are dominant on the opposite side. Two highly conserved glutamate residues, Glu^116^ and Glu^118^, are responsible for this negative charge. Each monomer has a deep pocket on its surface. In the trimeric structure, the depth of each pocket is limited by the β5 strand of the adjacent monomer (Figure 1C,D).

### Structural comparison of *S. aureus* YabJ with other proteins

**Figure 2:**
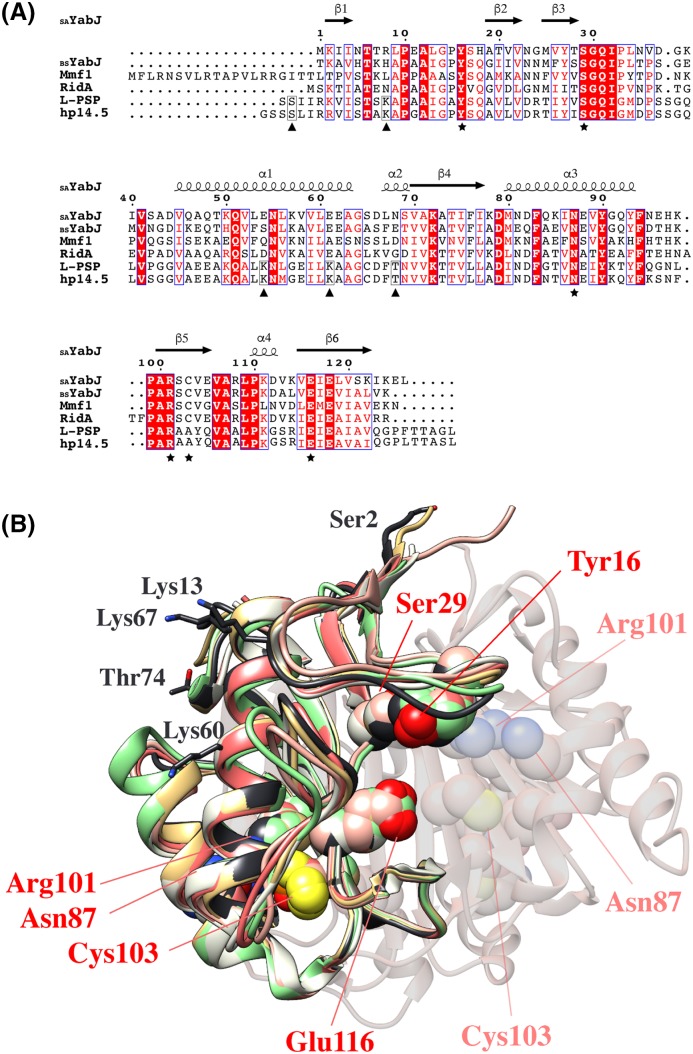
Comparison of _SA_YabJ with other members of the RidA subfamily (**A**) Comparison of _SA_YabJ with other members of the RidA subfamily. Sequence alignment of _SA_YabJ with YabJ from *B. subtilis* (59% sequence identity), Mmf1 from *S. cerevisiae* (42% sequence identity), RidA from *E. coli* (50% sequence identity), L-PSP from rat (38% sequence identity), and hp14.5 from human (42% sequence identity). Identical residues are colored white on a red background and similar residues are red on a white background. Secondary-structure elements (springs, α-helices; arrows, β-strands) are represented above and the sequences are numbered. The six footprint residues are marked as (★). The residues enclosed with black boxes in L-PSP and hp14.5 are expected to go through PTM and additionally marked as (▲). The figure was constructed using ESPript (http://espript.ibcp.fr) [73]. (**B**) The superposition of _SA_YabJ (pink) with the structures of YabJ from *B. subtilis* (salmon, 0.7 Å RMSD, PDB code: 1QD9), Mmf1 from *S. cerevisiae* (lime, 1.1 Å RMSD, PDB code: 3QUW), RidA from *E. coli* (green, 1.1 Å RMSD, PDB code: 1QU9), L-PSP from rat (black, 1.3 Å RMSD, PDB code: 1QAH), and hp14.5 from human (gold, 1.0 Å RMSD, PDB code: 1ONI). Six footprint residues are converged in the pocket and indicated as pink. The same residues from adjacent chains are shown in light pink. The residues that are expected to undergo PTMs in L-PSP are all oriented toward the solvent area and denoted in black.

The overall fold of _SA_YabJ shows high structural similarity to other members of the YjgF family [46–51]. _SA_YabJ is classified as a member of the typical RidA subfamily by sequence analysis, and all six footprint residues (Tyr^16^, Ser^29^, Asn^87^, Arg^101^, Cys^103^, and Glu^116^) are conserved. The absolutely conserved arginine (Arg^101^ in _SA_YabJ) in RidA and Rid1–Rid3 subfamilies has proven to be crucial for the catalysis of imine hydrolysis subfamilies. This arginine is not conserved in the members of Rid4–Rid7 subfamilies, and imine hydrolysis is not catalyzed by these proteins [9]. In the crystal structure of _SA_YabJ, all six footprint residues are positioned toward the deep pocket on the surface, where they are known to be involved in ligand binding (Figure 2). In the co-crystal structures of 2-oxobutanoate-bound *E. coli* TdcF and pyruvate-bound *Arabidopsis thaliana* RidA, ligands are co-ordinated in these pockets, interacting with arginine, cysteine, serine, and glutamate residues [52,53].

The _SA_YabJ structure was submitted to the DALI server (http://ekhinda.biocenter.helsinki.fi/dali_server) to obtain structural homologs [54]. Most structural matches were from members of the YjgF family, including RidA subfamily proteins YabJ from *B. subtilis* (Z-score = 24.7, 0.77 r.m.s.d., 124 equivalent Cα, 58% sequence identity), Mmf1 from *S. cerevisiae* (Z-score = 23.8, 0.85 r.m.s.d., 125 equivalent Cα, 40% sequence identity), RidA from *E. coli* (Z-score = 22.1, 1.05 r.m.s.d., 122 equivalent Cα, 48% sequence identity), L-PSP from *R. norvegicus* (Z-score = 22.1, 1.26 r.m.s.d., 125 equivalent Cα, 38% sequence identity), and hp14.5 from *Homo sapiens* (Z-score = 22.0, 1.00 r.m.s.d., 125 equivalent Cα, 42% sequence identity). Other matches from different Rid subfamilies include TdcF from *E. coli* (Z-score = 22.1, 1.12 r.m.s.d., 123 equivalent Cα, 51% sequence identity), RutC from *E. coli* (Z-score = 20.8, 1.20 r.m.s.d., 116 equivalent Cα, 38% sequence identity), and YoaB from *Salmonella typhimurium* (Z-score = 16.8, 1.35 r.m.s.d., 109 equivalent Cα, 30% sequence identity).

Since only mammalian RidA proteins are known to show nucleic acid-related activity, the structure is compared with mammalian L-PSP from rat and hp14.5 from human. For L-PSP from rat, one serine residue (Ser^2^), three lysine residues (Lys^13^, Lys^60^, and Lys^67^), and one threonine residue (Thr^74^) are expected to have PTMs, as acetylation, succinylation, and phosphorylation, respectively [55–57]. These five residues are all surface exposed and conserved in human hp14.5. Additionally, one of the six footprint residues, Cys^103^ in _SA_YabJ is replaced by alanine in both mammalian RidA proteins. This indicates that bacterial and mammalian RidA may perform different functions (Figure 2). At present, many structures from the YjgF family have been solved and most of them share the tightly packed trimeric structure mediated through the β-strands of each monomer. Despite the conserved quaternary structures throughout the YjgF family, several disparate metabolic functions are revealed. Therefore, detailed structural insights would produce significant contributions to our understanding of the biological functions of the YjgF family proteins.

### Chlorination of *S. aureus* YabJ

Proteins of the YjgF family are mostly highly acid-stable, evidenced by L-PSP and hp14.5, members of the RidA subfamily [49]. These proteins are prepared as soluble proteins in perchloric acid-treated rat liver and trichloroacetic acid extracts from human mononuclear phagocyte (MNP), respectively [19,20,57]. They both inhibit protein synthesis and L-PSP is also a ribonuclease. Interestingly, acid-extracted, native L-PSP, and hp14.5 show higher activity compared with their recombinant counterparts [20,58]. *E. coli* RidA shows chaperone activity after incubation with HOCl [5]. These previous studies show that chlorination plays a role in the physiology of RidA subfamily proteins. Protein chlorination causes alterations in protein structure via side chain or peptide bond amino group modification, and a corresponding functional change follows in several cases [59–61]. As other members of the RidA subfamily show high stability in acidic solution and chlorination, _SA_YabJ is predicted to be stable during chlorination. To study whether _SA_YabJ shows any functional changes upon chlorination, like *E. coli* RidA, chlorination of _SA_YabJ was performed.

**Figure 3:**
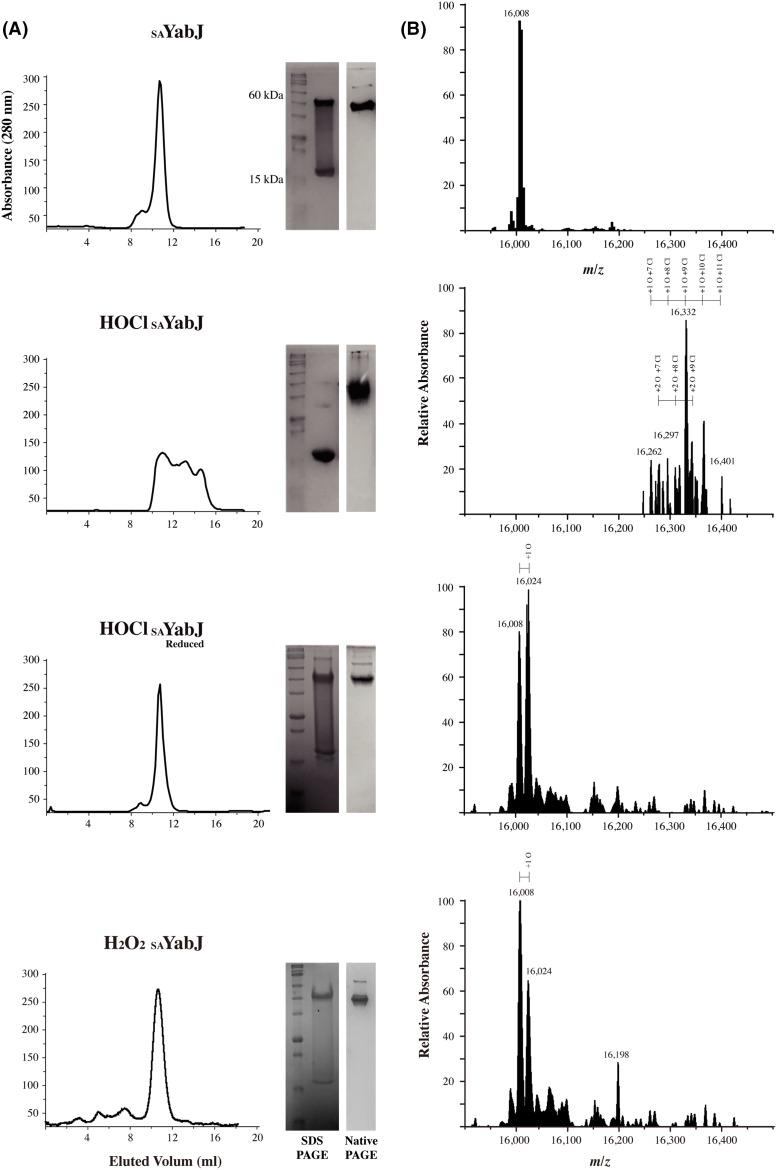
Chlorination of _SA_YabJ (**A**) The size-exclusion chromatography of native _SA_YabJ, HOCl-treated _SA_YabJ, reduced HOCl-treated _SA_YabJ with DTT, and H_2_O_2_-treated _SA_YabJ. Excess HOCl, DTT, and H_2_O_2_ were removed with extensive dialysis before size-exclusion chromatography. The right panel corresponds to SDS/PAGE (left) and native PAGE (right). Before chlorination, SDS/PAGE separates _SA_YabJ into two main bands, indicating trimer (50 kDa) and monomer (16 kDa). The single, intense peak in size-exclusion chromatography demonstrates the trimeric state of _SA_YabJ. When _SA_YabJ is treated with HOCl, three broadened peaks are observed and the trimer band at 50 kDa on SDS/PAGE disappeared. The SDS/PAGE of DTT-reduced, HOCl-treated _SA_YabJ shows a recovered trimer band and the single intense peak on size-exclusion chromatography re-appeared. H_2_O_2_-treated _SA_YabJ shows similar results: one single peak on size-exclusion chromatography, two separate bands on SDS/PAGE, while native PAGE shows a condensed band. (**B**) Mass spectroscopy of native and chemical-treated _SA_YabJ in the same order as (A). A major peak was observed at 16008 Da in native _SA_YabJ spectrum, corresponding to the calculated mass of _SA_YabJ. After the treatment of HOCl, a major peak was observed at 16332 Da, in addition to multiple peaks with ~35.45 Da intervals, indicating discreet additions of chloride ions. Another set of peaks with ~35.45 Da intervals is observed, implying simultaneous oxidation and chlorination occurs. Two main peaks with an approximate 16 Da mass difference appear when the HOCl-treated _SA_YabJ is reduced, indicating most chlorinated residues are reduced but some oxidized residues are not reduced. A similar spectrum is observed for H_2_O_2_-treated _SA_YabJ.

The chlorination state in solution is monitored by size-exclusion chromatography, PAGE, and mass spectroscopy. A single, intense peak with a molecular mass of ~50 kDa in size-exclusion chromatography indicates the presence of homogeneous _SA_YabJ trimer. Native PAGE shows a single band, although SDS/PAGE separates _SA_YabJ into two bands at 50 and 16 kDa, indicating trimer and monomer. Because the chemical detergent SDS affects protein oligomeric state, trimeric structure is dissociated during SDS/PAGE. After 30 min of chlorination at room temperature, a single peak splits and broadens in size-exclusion chromatography. This result corroborates the native PAGE, which shows a thick, spread band. SDS/PAGE indicates only one band at the monomer position. The chlorination propensity of _SA_YabJ was reversible. When the chlorinated _SA_YabJ is reduced with 10 mM DTT, the same result as native _SA_YabJ is observed. This chlorinated and reduced _SA_YabJ recovers a single intense peak in size-exclusion chromatography, trimeric conformation in SDS/PAGE, and the spread band on native PAGE becomes condensed. To discriminate the effects of chlorination from oxidation, the same experiment was conducted on oxidized _SA_YabJ. A single peak is observed from size-exclusion chromatography and PAGE shows a similar result to _SA_YabJ or chlorinated and reduced _SA_YabJ (Figure 3A). The chlorination state was also identified using mass spectroscopy. Based on the calculated mass of recombinant _SA_YabJ of 16008.9 Da, a single peak at 16008 Da corresponds to native _SA_YabJ. On the other hand, after chlorination, the major peak is shifted to the right, indicating that several amino acids of _SA_YabJ are covalently modified. Oxidation occurs simultaneously during chlorination with HClO, and a number of peaks appeared in the mass spectrum. The major peaks show intervals of ~35.45 Da, which correspond to the addition of multiple chlorine atoms. After reduction in chlorinated _SA_YabJ, chlorinated residues are reduced but oxidized residues remain. Mass spectra of chlorinated and reduced _SA_YabJ and oxidized _SA_YabJ are similar, indicating two main peaks with a mass difference of ~16 Da (Figure 3B).

### Chaperone activity was not observed in *S. aureus* YabJ

**Figure 4:**
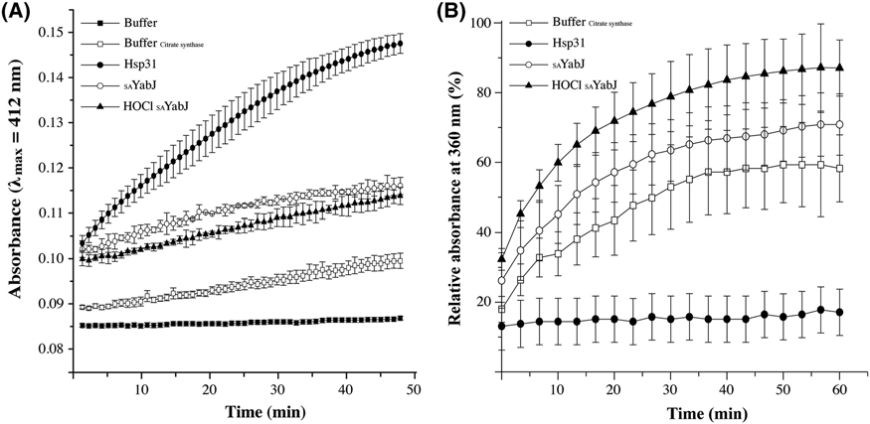
Chaperone activity of _SA_YabJ (**A**) The foldase chaperone activity was assayed by measuring chaperone-facilitated renaturation of citrate synthase. The buffer (▓) contained 50 μl of reaction mixture only (1 mM DTNB, 0.2 mM MnCl_2_, 0.4 mM oxaloacetic acid, 0.3 mM acetyl-CoA, and 100 mM Tris/HCl pH 8.0). The buffer with citrate synthase (□) contained additional 0.75 μg of denatured citrate synthase. Hsp31 (●) was used for the chaperone reference [41]. No chaperone activity was observed with _SA_YabJ before (○) and after (▲) chlorination. (**B**) Aggregation prevention assay. Aggregation of citrate synthase was measured by light scattering at 30°C. The citrate synthase was observed alone in a buffer (□), or mixed with 5 μM _SA_YabJ before (○) and after (▲) chlorination. Hsp31 that was used for the positive control (●) inhibits aggregation. Values are the mean of three separate determinations.

**Figure 5:**
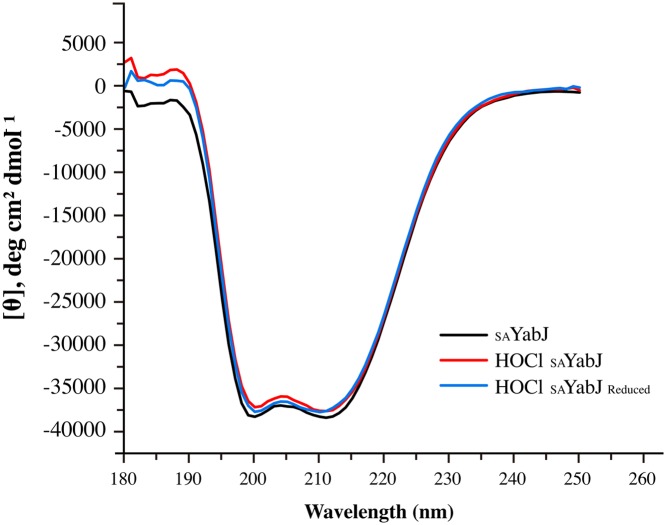
Secondary structure profile of _SA_YabJ Far-UV CD spectra of _SA_YabJ (black), HOCl-treated _SA_YabJ (red), and reduced HOCl-treated _SA_YabJ (blue). The spectra were nearly identical, indicating that chlorination does not affect the secondary structure of _SA_YabJ.

Chaperones are a functionally related group of proteins that assist in protein folding. Depending on the molecular weight, chaperones are classified into subfamilies. When the molecular weight is 34 kDa or less, they are called small heat shock proteins (sHSP) [62]. sHSP are often predicted to undergo dynamic assembly into oligomers, and perform chaperone activities by interacting with substrates [63]. Regulation is essential for ensuring proper physiological activity of these chaperones, and structural changes due to PTM are crucial for controlling their interactions with target proteins [64]. For example, Hsp33 undergoes structural rearrangement and is activated as a chaperone after exposure of HOCl [4]. A 20-kDa DJ-1 exhibits enhanced activity toward α-synuclein when its cysteine is oxidized to Cys-SO_2_H [65]. From our previous study, the 18-kDa SAV1875 lost its chaperone activity after its cysteine was fully oxidized from Cys-SO_2_H to Cys-SO_3_H [66]. Also, *E. coli* RidA shows chlorination-induced aggregation prevention chaperone activity [5]. Since _SA_YabJ is defined as a member of the RidA subfamily, displaying similar structural characteristics, chaperone activity was expected by chlorination in the same manner as *E. coli* RidA. However, both native and chlorinated _SA_YabJ do not show chaperone function in respects of the foldase activity or aggregation prevention activity (Figure 4). This result is surprising, considering the high degree of sequence and structural similarity between _SA_YabJ and *E. coli* RidA; both sharing 50% homology, comprising trimeric structures with an RMSD value of 1.1 Å, and displaying similar surface structures and charge distributions. Size-exclusion chromatography predicts *E. coli* RidA to be multimerized after the chlorination, but _SA_YabJ does not seem to form any higher oligomer (Figure 3A). Although the trimeric structure is supposed to be dissociated, _SA_YabJ was highly stable after chlorination. In addition, CD revealed that the secondary structure of dominant α-helices is well preserved after chlorination (Figure 5). Therefore, trimeric structure or higher oligomeric state might be the requisite to the chaperone activity of RidA subfamily proteins.

### Ribonuclease activity of *S. aureus* YabJ

The overall fold and trimeric structure of _SA_YabJ are well conserved throughout YjgF family members. However, the gene distribution of *yabJ* in *Staphylococcus* and *Bacillus* species is unusual [16]. Only *Staphylococcus* and *Bacillus* species have the *yabJ-spoVG* operon that encodes YjgF family protein YabJ and multifunctional protein SpoVG. The inactivation of the *yabJ-spoVG* operon produces strong attenuation of nuclease, protease, and lipase function. The regulation of this operon is mainly associated with SpoVG rather than YabJ, and the strong-binding affinity of SpoVG and nucleic acids is verified [14,16]. Although there are known functions of _BS_YabJ, including deamination and repression of *purA* transcription, detailed studies on the function of YabJ with regard to nucleic acids are limited [18]. From the gene clustering information, previous functional studies, and nucleic acid-related studies with L-PSP and hp14.5, _SA_YabJ is predicted to have ribonuclease activity. However, native _SA_YabJ did not cleave any nucleic acid. Since L-PSP from perchloric acid-treated rat liver reveals four times higher ribonuclease activity compared with recombinant L-PSP and hp14.5 extracted from trichloroacetic acid shows higher protein synthesis inhibition activity than the recombinant hp14.5, chlorination is speculated to affect the interaction with nucleic acids [19,20].

**Figure 6:**
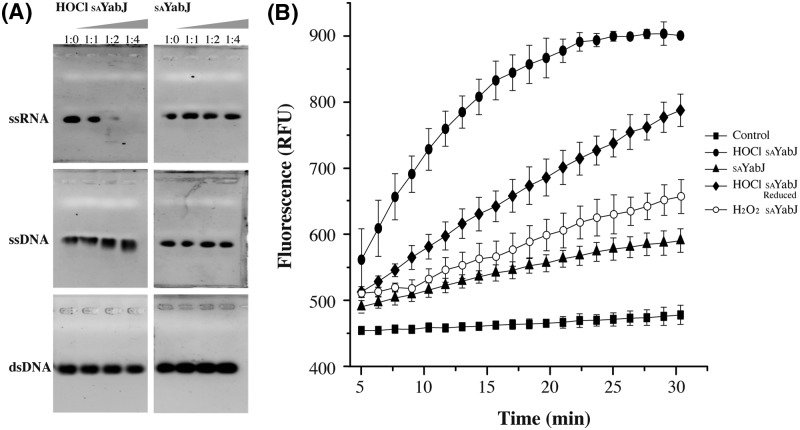
Ribonuclease activity of _SA_YabJ (**A**) Nucleic acids (ssRNA, ssDNA, and dsDNA) digestion activity experiments were analyzed by 0.8% agarose gel electrophoresis. A constant concentration of nucleic acids (30 μM) and an increasing concentration (0, 30, 60, and 120 μM) of _SA_YabJ and chlorinated _SA_YabJ were used. (**B**) Ribonuclease activity of _SA_YabJ was measured by a fluorescence-quenching assay. Fluorescent substrates were incubated with _SA_YabJ (▲), chlorinated _SA_YabJ (●), reduced chlorinated _SA_YabJ (♦), and oxidized _SA_YabJ (○). The control (▓) was incubated with buffer containing 50 mM Tris/HCl, pH 7.5, 150 mM NaCl. Each experiment was performed in triplicate.

Chlorinated _SA_YabJ was tested for ribonuclease activity. On gel electrophoresis, chlorinated _SA_YabJ displays the highest cleavage activity against ssRNA molecules and weak activity on ssDNA. However, chlorinated _SA_YabJ does not cleave dsDNA. The specificity on ssRNA corresponds previous observations in mammalian RidA proteins [19]. Native _SA_YabJ does not show nuclease activity toward any type of nucleic acid (Figure 6A). As chlorination is reversible, ribonuclease activity of chlorinated _SA_YabJ decreases after reduction. _SA_YabJ that was reduced by DTT after chlorination was no longer able to degrade ssRNA on gel electrophoresis (data not shown). The fluorescence-quenching assay using random RNA corroborates the results of gel electrophoresis. Chlorinated _SA_YabJ showed a significantly higher initial rate of RNA cleavage compared with native or oxidized _SA_YabJ. DTT-reduction after chlorination subsequently reduced this gain in activity of _SA_YabJ, showing that chlorination, not oxidation, is essential for the ribonuclease activity of _SA_YabJ (Figure 6B).

### Structural insights into the interactions of chlorinated *S. aureus* YabJ with nucleic acids

Although _SA_YabJ shows an uncertain oligomeric state after chlorination, chlorinated _SA_YabJ was highly stable at high concentrations at room temperature. Although CD also revealed that the secondary structure was maintained, the protein did not crystallize after chlorination. Since ribonuclease activity is not observed in most RidA family proteins, there is a lack of studies that have focussed the ribonuclease function and structure relationship of RidA. A notable feature in the RidA monomer structure is a groove that forms a pocket when it becomes a trimer. The diameter of this groove is ~20 Å, which is a sufficient distance to accommodate nucleic acids. The surface electrostatic potential distribution of the groove shows a positively charged surface patch comprising Lys^78^, Lys^111^, and Arg^108^. The _SA_YabJ monomer structure implies possible interactions with nucleic acids physically and chemically.

**Figure 7:**
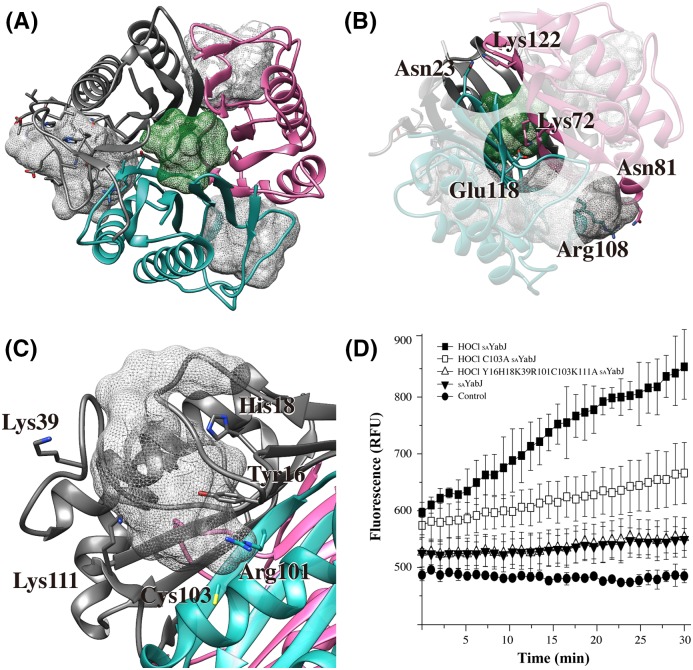
Binding pockets and pocket-forming sites residues identification (**A**) MetaPocket 2.0 found four pockets including the hole in the center of the trimer (green mesh) and a pocket on each monomer (gray meshes) of _SA_YabJ that are potential binding sites of substrates. (**B**) Three charge–charge interactions on the trimeric interface. (**C**) Detailed view of the one of the monomer pockets. The residues that can be chlorinated in the monomer pocket are labeled. (**D**) Ribonuclease activity assay was monitored in chlorinated _SA_YabJ (▓), chlorinated C103A _SA_YabJ mutant (□), chlorinated pocket-forming sites mutant (Y16H18K39R101C103K111A) (△), and _SA_YabJ before chlorination (▼). The data from three scans were averaged.

The possible binding pocket of nucleic acids is detectable from the native _SA_YabJ crystal structure. To identify the location of the pocket, the _SA_YabJ structure was submitted to MetaPocket 2.0 (http://projects.biotec.tu-dresden.de/metapocket/) [67,68]. Four putative pockets are detected, including the hole in the center area and the pocket on each monomer’s surface (Figure 7A). Ligand-bound crystal structures of other YjgF family members, *E. coli* TdcF, *A. thaliana* RidA, and human hp14.5, show that ligands are found in the pocket on the monomer’s surface [52,53]. These studies suggest that the pocket on the monomer could be the _SA_YabJ binding site for nucleic acids.

Amino acids that can be easily modified after HOCl treatment are sulphur-containing cysteine and methionine, or amide-containing arginine, histidine, and lysine. In addition, other residues, such as tyrosine and tryptophan, are known to become chlorinated [69,70]. For example, tyrosine is chlorinated to 3-chlorotyrosine and chlorination of lysine can form monochloramines or dichloramines [69,71,72]. Lys^72^, Asn^81^, and Lys^122^ interact with adjacent subunit residues (Glu^118^, Arg^108^, and Asn^23^, respectively) through charge–charge interaction on the trimeric interface (Figure 7B). When _SA_YabJ is chlorinated, these residues might be affected by chlorination and the trimeric state would be destabilized. Residues Tyr^16^, His^18^, Lys^39^, Arg^101^, Cys^103^, and Lys^111^ are in the flexible loop region and responsible for pocket formation. These residues are protruded toward the pocket and may be responsible for the altered characteristics following chlorination (Figure 7C). To study the effect of these residues, we performed an additional ribonuclease activity assay using chlorinated C103A _SA_YabJ mutant and chlorinated pocket-forming sites mutant (Y16H18K39R101C103K111A). The fluorescence intensity was reduced in C103A _SA_YabJ, indicating Cys^103^ performs an important role. The pocket-forming sites mutant lost ribonuclease activity (Figure 7D). On the basis of the study, we suggest that residues around monomer pocket are important for chlorinated _SA_YabJ performing ribonuclease activity.

## Discussion

Bacterial cells are prone to be exposed to a chlorinating milieu. Not only from household bleach, HOCl is produced during a host’s phagocytic immune reaction, mainly from macrophages and neutrophils. HOCl is a highly reactive component, reacting with multiple chemical entities, such as proteins and fatty acids, and lead to suppression of DNA synthesis [6–8,71]. However, successful pathogens resist the host cellular defense as a function of structural or biochemical properties. Some proteins are modified and retained/converted their function under chlorination. For example, Hsp33, RidA, and DUK114 gain chaperone activity upon chlorination. Even though chlorination plays one of the most important roles in microbial killing, little is known about chlorination and bacterial response [4,5,12].

In the present study, we elucidate the structure and chlorination-related function of _SA_YabJ as a member of the YjgF family. The crystal structure of _SA_YabJ shows a homologous fold and trimeric structure, which are the pertinent traits of the YjgF family. Each monomer has a deep pocket on their surface, and there is a hole at the trimeric center. The presence of trimeric structure, pockets, and central hole are well conserved in other members of the YjgF family. The six footprint residues, which are used to differentiate YjgF subfamilies, are on a hole and pockets, implying the importance of structural features. Specifically, the amino acid composition of the pockets and hole structure of _SA_YabJ are similar to those of YabJ from *B. subtilis*. In addition, _SA_YabJ and _BS_YabJ share the same genetic array: the *yabJ-spoVG* gene cluster, which suggests that they might perform the same function. However, functional studies of _BS_YabJ are limited to deamination and inhibition of *purA* transcription.

We first identified _SA_YabJ as a ribonuclease that is activated after chlorination. Native _SA_YabJ did not show any ribonucleolytic activity. After chlorination, _SA_YabJ digests ssRNA efficiently, and also cleaves ssDNA to a lesser extent, but loses the activity when reduced. The loss of activity is due to the reduction in chlorinated _SA_YabJ as the difference was confirmed by size-exclusion chromatography and SDS/Native PAGE. In addition, we revealed that this gain of ribonuclease activity is specifically due to chlorination, not oxidation. This finding matches the fact that native L-PSP and hp14.5 show higher activity compared with recombinant proteins. The native L-PSP and hp14.5 were extracted using perchloric acid and trichloroacetic acid, respectively; conditions that permit chlorination of the proteins. Recombinant L-PSP and hp14.5 were, however, expressed in *E. coli*, and purified in buffers where chlorination was less likely.

This raises the question of exactly what modifications lead to _SA_YabJ gaining ribonucleolytic activity following chlorination. Mass spectroscopy shows an increase in more than 300 Da after chlorination, indicating that ~11 amino acids can be affected from the protein’s 126 amino residues. The result illustrates that chlorination and oxidation occur concurrently and various residues are influenced besides the sulphur-containing residues, methionine, and cysteine, as there are only four of these in total. From the mutants study, we have shown that Tyr^16^, His^18^, Lys^39^, Arg^101^, Cys^103^, and Lys^111^, which are predicted as the potential nucleic acid binding groove, are important for the chlorinated _SA_YabJ performing ribonuclease activity. Further investigation into the chlorination and activation of YabJ would further our understanding of the fundamentals of the chlorination PTM. In addition, the present study has led to the novel discovery of the chlorination-induced ribonuclease function of YabJ.

## Accession number

Protein co-ordinate and structure factor have been deposited in the RCSB PDB under code 5YU2.

## Abbreviations

DTNB: 5,5′-dithiobis(2-nitrobenzoic acid)
LIC: ligation-independent cloning
L-PSP: liver perchloric acid-soluble protein
PTM: post-translational modification
sHSP: small heat shock protein
TEV: tobacco etch virus
TBE: Tris-borate-EDTA
YjgF family: YjgF/YER057c/UK114 family

## References

[1] Karve T.M. and Cheema A.K. (2011) Small changes huge impact: the role of protein posttranslational modifications in cellular homeostasis and disease. J. Amino Acids 2011, 207691 10.4061/2011/207691 22312457 PMC3268018

[2] Duan G. and Walther D. (2015) The roles of post-translational modifications in the context of protein interaction networks. PLoS Comput. Biol. 11, e1004049 10.1371/journal.pcbi.1004049 25692714 PMC4333291

[3] Pan Z., Liu Z., Cheng H., Wang Y., Gao T., Ullah S. (2014) Systematic analysis of the *in situ* crosstalk of tyrosine modifications reveals no additional natural selection on multiply modified residues. Sci. Rep. 4, 7331 10.1038/srep07331 25476580 PMC4256647

[4] Winter J., Ilbert M., Graf P.C., Ozcelik D. and Jakob U. (2008) Bleach activates a redox-regulated chaperone by oxidative protein unfolding. Cell 135, 691–701 10.1016/j.cell.2008.09.024 19013278 PMC2606091

[5] Muller A., Langklotz S., Lupilova N., Kuhlmann K., Bandow J.E. and Leichert L.I. (2014) Activation of RidA chaperone function by N-chlorination. Nat. Commun. 5, 5804 10.1038/ncomms6804 25517874 PMC4284807

[6] Gray M.J., Wholey W.Y. and Jakob U. (2013) Bacterial responses to reactive chlorine species. Annu. Rev. Microbiol. 67, 141–160 10.1146/annurev-micro-102912-142520 23768204 PMC3891400

[7] Hurst J.K. (2012) What really happens in the neutrophil phagosome? Free Radic. Biol. Med. 53, 508–520 22609248 10.1016/j.freeradbiomed.2012.05.008 PMC4382085

[8] Klebanoff S.J. (2005) Myeloperoxidase: friend and foe. J. Leukoc. Biol. 77, 598–625 10.1189/jlb.1204697 15689384

[9] Niehaus T.D., Gerdes S., Hodge-Hanson K., Zhukov A., Cooper A.J., ElBadawi-Sidhu M. (2015) Genomic and experimental evidence for multiple metabolic functions in the RidA/YjgF/YER057c/UK114 (Rid) protein family. BMC Genomics 16, 382 10.1186/s12864-015-1584-3 25975565 PMC4433059

[10] Lambrecht J.A., Flynn J.M. and Downs D.M. (2012) Conserved YjgF protein family deaminates reactive enamine/imine intermediates of pyridoxal 5′-phosphate (PLP)-dependent enzyme reactions. J. Biol. Chem. 287, 3454–3461 10.1074/jbc.M111.304477 22094463 PMC3270999

[11] Lambrecht J.A., Browne B.A. and Downs D.M. (2010) Members of the YjgF/YER057c/UK114 family of proteins inhibit phosphoribosylamine synthesis *in vitro*. J. Biol. Chem. 285, 34401–34407 10.1074/jbc.M110.160515 20817725 PMC2966054

[12] Farkas A., Nardai G., Csermely P., Tompa P. and Friedrich P. (2004) DUK114, the *Drosophila* orthologue of bovine brain calpain activator protein, is a molecular chaperone. Biochem. J. 383, 165–170 10.1042/BJ20040668 15250825 PMC1134055

[13] Hanson A.D., Pribat A., Waller J.C. and de Crecy-Lagard V. (2009) ‘Unknown’ proteins and ‘orphan’ enzymes: the missing half of the engineering parts list - and how to find it. Biochem. J. 425, 1–11 10.1042/BJ20091328 20001958 PMC3022307

[14] Schulthess B., Meier S., Homerova D., Goerke C., Wolz C., Kormanec J. (2009) Functional characterization of the sigmaB-dependent *yabJ-spoVG* operon in *Staphylococcus aureus*: role in methicillin and glycopeptide resistance. Antimicrob. Agents Chemother. 53, 1832–1839 10.1128/AAC.01255-08 19223635 PMC2681525

[15] Van Schaik W. and Abee T. (2005) The role of sigmaB in the stress response of Gram-positive bacteria - targets for food preservation and safety. Curr. Opin. Biotechnol. 16, 218–224 10.1016/j.copbio.2005.01.008 15831390

[16] Schulthess B., Bloes D.A., Francois P., Girard M., Schrenzel J., Bischoff M. (2011) The sigmaB-dependent *yabJ-spoVG* operon is involved in the regulation of extracellular nuclease, lipase, and protease expression in *Staphylococcus aureus*. J. Bacteriol. 193, 4954–4962 10.1128/JB.05362-11 21725011 PMC3165683

[17] Meier S., Goerke C., Wolz C., Seidl K., Homerova D., Schulthess B. (2007) sigmaB and the sigmaB-dependent *arlRS* and *yabJ-spoVG* loci affect capsule formation in *Staphylococcus aureus*. Infect. Immun. 75, 4562–4571 10.1128/IAI.00392-07 17635871 PMC1951174

[18] Rappu P., Shin B.S., Zalkin H. and Mantsala P. (1999) A role for a highly conserved protein of unknown function in regulation of *Bacillus subtilis purA* by the purine repressor. J. Bacteriol. 181, 3810–3815 10368157 10.1128/jb.181.12.3810-3815.1999 PMC93860

[19] Morishita R., Kawagoshi A., Sawasaki T., Madin K., Ogasawara T., Oka T. (1999) Ribonuclease activity of rat liver perchloric acid-soluble protein, a potent inhibitor of protein synthesis. J. Biol. Chem. 274, 20688–20692 10.1074/jbc.274.29.20688 10400702

[20] Schmiedeknecht G., Kerkhoff C., Orso E., Stohr J., Aslanidis C., Nagy G.M. (1996) Isolation and characterization of a 14.5-kDa trichloroacetic-acid-soluble translational inhibitor protein from human monocytes that is upregulated upon cellular differentiation. Eur. J. Biochem. 242, 339–351 10.1111/j.1432-1033.1996.0339r.x 8973653

[21] Aslanidis C. and de Jong P.J. (1990) Ligation-independent cloning of PCR products (LIC-PCR). Nucleic Acids Res. 18, 6069–6074 10.1093/nar/18.20.6069 2235490 PMC332407

[22] Jeong J.Y., Yim H.S., Ryu J.Y., Lee H.S., Lee J.H., Seen D.S. (2012) One-step sequence- and ligation-independent cloning as a rapid and versatile cloning method for functional genomics studies. Appl. Environ. Microbiol. 78, 5440–5443 10.1128/AEM.00844-12 22610439 PMC3416421

[23] Eschenfeldt W.H., Lucy S., Millard C.S., Joachimiak A. and Mark I.D. (2009) A family of LIC vectors for high-throughput cloning and purification of proteins. Methods Mol. Biol. 498, 105–115 10.1007/978-1-59745-196-3_7 18988021 PMC2771622

[24] Otwinowski Z. and Minor W. (1997) Processing of X-ray diffraction data collected in oscillation mode. Methods Enzymol. 276, 307–326 10.1016/S0076-6879(97)76066-X 27754618

[25] Winn M.D., Ballard C.C., Cowtan K.D., Dodson E.J., Emsley P., Evans P.R. (2011) Overview of the CCP4 suite and current developments. Acta Crystallogr. D Biol. Crystallogr. 67, 235–242 10.1107/S0907444910045749 21460441 PMC3069738

[26] Vagin A. and Teplyakov A. (2010) Molecular replacement with MOLREP. Acta Crystallogr. D Biol. Crystallogr. 66, 22–25 10.1107/S0907444909042589 20057045

[27] Emsley P. and Cowtan K. (2004) Coot: model-building tools for molecular graphics. Acta Crystallogr. D Biol. Crystallogr. 60, 2126–2132 10.1107/S0907444904019158 15572765

[28] Murshudov G.N., Skubak P., Lebedev A.A., Pannu N.S., Steiner R.A., Nicholls R.A. (2011) REFMAC5 for the refinement of macromolecular crystal structures. Acta Crystallogr. D Biol. Crystallogr. 67, 355–367 10.1107/S0907444911001314 21460454 PMC3069751

[29] Murshudov G.N., Vagin A.A. and Dodson E.J. (1997) Refinement of macromolecular structures by the maximum-likelihood method. Acta Crystallogr. D Biol. Crystallogr. 53, 240–255 10.1107/S0907444996012255 15299926

[30] Adams P.D., Afonine P.V., Bunkoczi G., Chen V.B., Davis I.W., Echols N. (2010) PHENIX: a comprehensive python-based system for macromolecular structure solution. Acta Crystallogr. D Biol. Crystallogr. 66, 213–221 10.1107/S0907444909052925 20124702 PMC2815670

[31] Afonine P.V., Grosse-Kunstleve R.W., Echols N., Headd J.J., Moriarty N.W., Mustyakimov M. (2012) Towards automated crystallographic structure refinement with phenix.refine.. Acta Crystallogr. D Biol. Crystallogr. 68, 352–367 10.1107/S0907444912001308 22505256 PMC3322595

[32] Headd J.J., Echols N., Afonine P.V., Grosse-Kunstleve R.W., Chen V.B., Moriarty N.W. (2012) Use of knowledge-based restraints in phenix.refine to improve macromolecular refinement at low resolution. Acta Crystallogr. D Biol. Crystallogr. 68, 381–390 10.1107/S0907444911047834 22505258 PMC3322597

[33] Brunger A.T. (1992) Free R value: a novel statistical quantity for assessing the accuracy of crystal structures. Nature 355, 472–475 10.1038/355472a0 18481394

[34] Laskowski R.A., Rullmannn J.A., MacArthur M.W., Kaptein R. and Thornton J.M. (1996) AQUA and PROCHECK-NMR: programs for checking the quality of protein structures solved by NMR. J. Biomol. NMR 8, 477–486 10.1007/BF00228148 9008363

[35] Pettersen E.F., Goddard T.D., Huang C.C., Couch G.S., Greenblatt D.M., Meng E.C. (2004) UCSF Chimera - a visualization system for exploratory research and analysis. J. Comput. Chem. 25, 1605–1612 10.1002/jcc.20084 15264254

[36] Krissinel E. and Henrick K. (2007) Inference of macromolecular assemblies from crystalline state. J. Mol. Biol. 372, 774–797 10.1016/j.jmb.2007.05.022 17681537

[37] Laemmli U.K. (1970) Cleavage of structural proteins during the assembly of the head of bacteriophage T4. Nature 227, 680–685 10.1038/227680a0 5432063

[38] Greenfield N.J. (2006) Using circular dichroism spectra to estimate protein secondary structure. Nat. Protoc. 1, 2876–2890 10.1038/nprot.2006.202 17406547 PMC2728378

[39] Lee G.J. (1995) Assaying proteins for molecular chaperone activity. Methods Cell Biol. 50, 325–334 10.1016/S0091-679X(08)61040-7 8531805

[40] Zhi W., Landry S.J., Gierasch L.M. and Srere P.A. (1992) Renaturation of citrate synthase: influence of denaturant and folding assistants. Protein Sci. 1, 522–529 10.1002/pro.5560010407 1363914 PMC2142213

[41] Kim H.J., Lee K.Y., Kwon A.R. and Lee B.J. (2017) Structural and functional studies of SAV0551 from *Staphylococcus aureus* as a chaperone and glyoxalase III. Biosci. Rep. 37, 6 10.1042/BSR20171106 PMC5691139 29046369

[42] Morgunov I. and Srere P.A. (1998) Interaction between citrate synthase and malate dehydrogenase. Substrate channeling of oxaloacetate. J. Biol. Chem. 273, 29540–29544 10.1074/jbc.273.45.29540 9792662

[43] Eyraud A., Tattevin P., Chabelskaya S. and Felden B. (2014) A small RNA controls a protein regulator involved in antibiotic resistance in *Staphylococcus aureus*. Nucleic Acids Res. 42, 4892–4905 10.1093/nar/gku149 24557948 PMC4005690

[44] Park C., Kelemen B.R., Klink T.A., Sweeney R.Y., Behlke M.A., Eubanks S.R. (2001) Fast, facile, hypersensitive assays for ribonucleolytic activity. Methods Enzymol. 341, 81–94 10.1016/S0076-6879(01)41146-3 11582813

[45] Kelemen B.R., Klink T.A., Behlke M.A., Eubanks S.R., Leland P.A. and Raines R.T. (1999) Hypersensitive substrate for ribonucleases. Nucleic Acids Res. 27, 3696–3701 10.1093/nar/27.18.3696 10471739 PMC148625

[46] Volz K. (1999) A test case for structure-based functional assignment: the 1.2 Å crystal structure of the yjgF gene product from *Escherichia coli*. Protein Sci. 8, 2428–2437 10.1110/ps.8.11.2428 10595546 PMC2144179

[47] Knapik A.A., Petkowski J.J., Otwinowski Z., Cymborowski M.T., Cooper D.R., Chruszcz M. (2012) Structure of *Escherichia coli* RutC, a member of the YjgF family and putative aminoacrylate peracid reductase of the *rut* operon. Acta Crystallogr. Sect. F Struct. Biol. Cryst. Commun. 68, 1294–1299 10.1107/S1744309112041796 23143235 PMC3515367

[48] Burman J.D., Stevenson C.E., Sawers R.G. and Lawson D.M. (2007) The crystal structure of *Escherichia coli* TdcF, a member of the highly conserved YjgF/YER057c/UK114 family. BMC Struct. Biol. 7, 30 10.1186/1472-6807-7-30 17506874 PMC1884159

[49] Sinha S., Rappu P., Lange S.C., Mantsala P., Zalkin H. and Smith J.L. (1999) Crystal structure of *Bacillus subtilis* YabJ, a purine regulatory protein and member of the highly conserved YjgF family. Proc. Natl. Acad. Sci. U.S.A. 96, 13074–13079 10.1073/pnas.96.23.13074 10557275 PMC23902

[50] Miyakawa T., Lee W.C., Hatano K., Kato Y., Sawano Y., Miyazono K. (2006) Crystal structure of the YjgF/YER057c/UK114 family protein from the hyperthermophilic archaeon *Sulfolobus tokodaii* strain 7. Proteins 62, 557–561 10.1002/prot.20778 16323205

[51] Thakur K.G., Praveena T. and Gopal B. (2010) *Mycobacterium tuberculosis* Rv2704 is a member of the YjgF/YER057c/UK114 family. Proteins 78, 773–778 19899170 10.1002/prot.22623 PMC3068300

[52] Manjasetty B.A., Delbruck H., Pham D.T., Mueller U., Fieber-Erdmann M., Scheich C. (2004) Crystal structure of *Homo sapiens* protein hp14.5. Proteins 54, 797–800 10.1002/prot.10619 14997576

[53] Liu X., Zeng J., Chen X. and Xie W. (2016) Crystal structures of RidA, an important enzyme for the prevention of toxic side products. Sci. Rep. 6, 30494 10.1038/srep30494 27458092 PMC4960622

[54] Holm L. and Rosenstrom P. (2010) Dali server: conservation mapping in 3D. Nucleic Acids Res. 38, W545–549 10.1093/nar/gkq366 20457744 PMC2896194

[55] Park J., Chen Y., Tishkoff D.X., Peng C., Tan M., Dai L. (2013) SIRT5-mediated lysine desuccinylation impacts diverse metabolic pathways. Mol. Cell. 50, 919–930 10.1016/j.molcel.2013.06.001 23806337 PMC3769971

[56] Bian Y., Song C., Cheng K., Dong M., Wang F., Huang J. (2014) An enzyme assisted RP-RPLC approach for in-depth analysis of human liver phosphoproteome. J. Proteomics 96, 253–262 10.1016/j.jprot.2013.11.014 24275569

[57] Oka T., Tsuji H., Noda C., Sakai K., Hong Y.M., Suzuki I. (1995) Isolation and characterization of a novel perchloric acid-soluble protein inhibiting cell-free protein synthesis. J. Biol. Chem. 270, 30060–30067 10.1074/jbc.270.50.30060 8530410

[58] Oka T., Nishimoto Y., Sasagawa T., Kanouchi H., Kawasaki Y. and Natori Y. (1999) Production of functional rat liver PSP protein in *Escherichia coli*. Cell Mol. Life Sci. 55, 131–134 10.1007/s000180050277 10065159 PMC11147094

[59] Curtis M.P. and Neidigh J.W. (2014) Kinetics of 3-nitrotyrosine modification on exposure to hypochlorous acid. Free Radic. Biol. Res. 48, 1355–1362 10.3109/10715762.2014.954110 25119650

[60] Gianazza E., Crawford J. and Miller I. (2007) Detecting oxidative post-translational modifications in proteins. Amino Acids 33, 51–56 10.1007/s00726-006-0410-2 17021655

[61] Chen H.J., Yang Y.F., Lai P.Y. and Chen P.F. (2016) Analysis of chlorination, nitration, and nitrosylation of tyrosine and oxidation of methionine and cysteine in hemoglobin from type 2 diabetes mellitus patients by nanoflow liquid chromatography tandem mass spectrometry. Anal. Chem. 88, 9276–9284 10.1021/acs.analchem.6b02663 27541571

[62] Sun Y. and MacRae T.H. (2005) Small heat shock proteins: molecular structure and chaperone function. Cell Mol. Life Sci. 62, 2460–2476 10.1007/s00018-005-5190-4 16143830 PMC11138385

[63] Mattoo R.U., Sharma S.K., Priya S., Finka A. and Goloubinoff P. (2013) Hsp110 is a bona fide chaperone using ATP to unfold stable misfolded polypeptides and reciprocally collaborate with Hsp70 to solubilize protein aggregates. J. Biol. Chem. 288, 21399–21411 10.1074/jbc.M113.479253 23737532 PMC3774407

[64] Cloutier P. and Coulombe B. (2013) Regulation of molecular chaperones through post-translational modifications: decrypting the chaperone code. Biochim. Biophys. Acta 1829, 443–454 10.1016/j.bbagrm.2013.02.010 23459247 PMC4492711

[65] Zhou W., Zhu M., Wilson M.A., Petsko G.A. and Fink A.L. (2006) The oxidation state of DJ-1 regulates its chaperone activity toward alpha-synuclein. J. Mol. Biol. 356, 1036–1048 10.1016/j.jmb.2005.12.030 16403519

[66] Kim H.J., Kwon A.R. and Lee B.J. (2016) Structural and functional insight into the different oxidation states of SAV1875 from *Staphylococcus aureus*. Biochem. J. 473, 55–66 10.1042/BJ20150256 26487697

[67] Huang B. (2009) MetaPocket: a meta approach to improve protein ligand binding site prediction. OMICS 13, 325–330 10.1089/omi.2009.0045 19645590

[68] Zhang Z., Li Y., Lin B., Schroeder M. and Huang B. (2011) Identification of cavities on protein surface using multiple computational approaches for drug binding site prediction. Bioinformatics 27, 2083–2088 10.1093/bioinformatics/btr331 21636590

[69] Pattison D.I. and Davies M.J. (2001) Absolute rate constants for the reaction of hypochlorous acid with protein side chains and peptide bonds. Chem. Res. Toxicol. 14, 1453–1464 10.1021/tx0155451 11599938

[70] Na C. and Olson T.M. (2007) Relative reactivity of amino acids with chlorine in mixtures. Environ. Sci. Technol. 41, 3220–3225 10.1021/es061999e 17539529

[71] Mohiuddin I., Chai H., Lin P.H., Lumsden A.B., Yao Q. and Chen C. (2006) Nitrotyrosine and chlorotyrosine: clinical significance and biological functions in the vascular system. J. Surg. Res. 133, 143–149 10.1016/j.jss.2005.10.008 16360172

[72] Ryan B.J., Nissim A. and Winyard P.G. (2014) Oxidative post-translational modifications and their involvement in the pathogenesis of autoimmune diseases. Redox Biol. 2, 715–724 10.1016/j.redox.2014.05.004 24955328 PMC4062766

[73] Robert X. and Gouet P. (2014) Deciphering key features in protein structures with the new ENDscript server. Nucleic Acids Res. 42, W320–324 10.1093/nar/gku316 24753421 PMC4086106

